# Validation of a Miniaturized Test Loop for the Assessment of Human Blood Damage by Continuous-Flow Left-Ventricular Assist Devices

**DOI:** 10.1007/s10439-021-02849-1

**Published:** 2021-08-24

**Authors:** Eva Woelke, Ilona Mager, Thomas Schmitz-Rode, Ulrich Steinseifer, Johanna C. Clauser

**Affiliations:** Department of Cardiovascular Engineering, Institute of Applied Medical Engineering, Helmholtz Institute, Medical Faculty, RWTH Aachen University, Pauwelsstr. 20, 52074 Aachen, Germany

**Keywords:** *In vitro* testing, Hemolysis, LVAD, Heartmate 3, von Willebrand factor, Hemocompatibility

## Abstract

Despite improved hemocompatibility of left-ventricular assist devices (LVADs), assessment of blood damage remains mandatory in preclinical testing as standardized by ASTM-F1841. The most relevant test fluid is fresh, non-pooled human blood, but the limited volume of a standard donation requires significantly smaller loops than those commonly used with animal blood. In a recent study with porcine blood, we verified a miniaturized test loop with only 160 mL for the ASTM-conform paired testing of at least two LVADs and a static reference. Here, we validated this mini test loop for standardized assessment of blood damage with one 450-mL single donation of fresh human blood. Blood damage was assessed for HeartMate 3 and BPX-80 in 9 experiments with heparinized human blood for 6 hours. We analyzed plasma free hemoglobin, von Willebrand factor (vWF) concentration and collagen-binding functionality and calculated indices of hemolysis and vWF-ratios. Overall, we observed less blood damage compared to our previous study; however, the differences in mean indices of hemolysis and in mean normalized vWF-ratio between BPX-80 and HeartMate 3 were consistent for human blood. Thus, our mini test loop proved to be valid for preclinical standardized assessment of blood damage with only 450 mL of fresh human blood.

## Introduction

Despite continuous optimization in the development of left-ventricular assist devices (LVADs), lower degrees of hemolysis or sub-lethal red blood cell damage,^20^ shear-dependent von Willebrand factor (vWF) degradation^21^ and platelet dysfunction^4^,^5^,^11^ still impact LVAD patients and can cumulatively trigger pathways that lead to hemorrhagic and ischemic adverse events.^12^,^14^,^23^ Thus, assessment of blood damage remains mandatory for preclinical LVAD testing.

Since 1997, the American Society of Testing and Materials (ASTM) standard F1841 regulates the *in vitro* assessment of hemolysis in continuous-flow LVADs to ensure equal testing at a clinically relevant operating point.^1^,^2^ Although the usage of bovine or porcine blood is a valid option, testing with fresh, non-pooled human blood will yield more relevant results.^7^ However, the limited volume of a single donation of fresh human blood requires significantly smaller test loops than the original ASTM standard loop commonly used with animal blood. Since the past few years, studies have started to address this issue,^3^–^5^,^17^,^29^ mostly using a test loop with a volume of 300 mL. However, paired testing of at least two LVADs and a static reference is mandatory for certification to overcome variabilities in donor blood and handling during testing and to monitor general blood damage over time at each test day, respectively. This requires an even further miniaturized test loop, especially regarding the elevated target hematocrit of 35 ± 2% of the newly approved ASTM F1841-19, that will be mandatory for preclinical LVAD testing from July 12th 2021 onwards.^2^

In a study with porcine blood, we recently verified a miniaturized test loop set-up of only 160 mL for standardized assessment of blood damage by continuous-flow LVADs, that allows for the paired testing of at least two LVADs and a static reference with 450 mL non-pooled blood.^27^

In this study, we validate our mini test loop for the assessment of hemolysis and vWF degradation at ASTM-conform operating point with one single donation of fresh human blood per paired test.

## Materials and Methods

In *n* = 9 independent experiments, we assessed the LVAD-related blood damage of the implantable continuous-flow LVAD HeartMate 3™ (HM3, Abbott, USA) and the extracorporeal BPX-80 Bio-Pump® (BPX-80, Medtronic, Ireland) with our recently described mini test loop.^27^ Analogous to the verification with porcine blood,^27^ the validation of the mini test loop with human blood was performed according to the ASTM F1841-97 (2017) standard.^1^

### Test Loop Set-up

**Figure 1:**
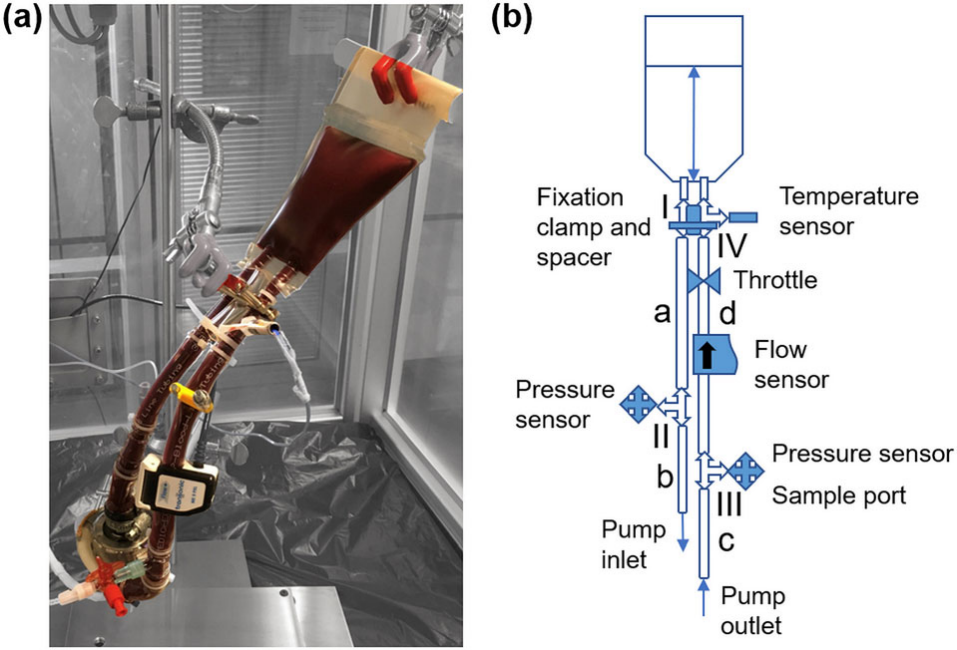
Set-up of mini test loop (a) and schematic (b) with tubing lengths *a* = 9 cm for both test loops, *b* =*c* = 6 cm for BPX-80 and *b* = *c* = 4 cm for HM3, *d* = 15 cm for both test loops; connectors I = IV = 1/4″–3/8″ for both test loops, II = III = 3/8″–3/8″ for BPX-80 and II = III = 1/2″–3/8″ for HM3 and reservoir lengths of 5.5 cm for BPX-80 and 9.5 cm for HM3. Part (b) is reprinted by permission from Springer Nature, Woelke *et al*.,^27^ Copyright 2019.

The mini test loops were set up as previously described.^27^ In brief, the mini test loops are downscaled to one-third (160 mL) of the ASTM F1841-97 (2017)^1^ priming volume, and include a throttle to yield the pressure head of 100 mmHg and mounted sensors for pressure (Xtrans, CODAN, Germany), temperature (Medos, Germany) and flow (Transonic, USA) (Fig. 1). To accommodate the size of the HM3 in- and outlet, 4 cm of tubing at each position are replaced with 1/2″-diameter PVC-tubing. To prevent bending of the tubing at the inlet and outlet of the mini test loop reservoir, connectors are fixed with a spacer and a clamp. Temperature of the mini test loops was controlled with heating-hoods.

### Blood Collection and Preparation

The study was approved by the local ethics committee (Ethical Approval Number EK141/20) and informed written consent was obtained from all donors. For each of the 9 tests, a single donation of 450 mL human blood (15,000 international units L^−1^ sodium heparin (B. Braun, Germany), 0.09% (w/v) glucose, 2.0% (v/v) isotonic saline solution, 0.016 g L^−1^ gentamycin) was freshly collected (Compoflex Transferbag T2131, Fresenius Kabi, Germany) from healthy male volunteers free from antiplatelet or anticoagulant medication for at least 10 days prior to donation. The blood was inspected for hematocrit, red and white blood cell and platelet count (hematology analyzer Sysmex XT2000i vet, Sysmex, Germany) and hemoglobin and lactate concentration (blood gas analyzer ABL 825 Flex, Radiometer, Germany). Blood dilution to the set hematocrit of 30 ± 2% was performed during priming and filling of each test loop by adding pre-calculated volumes of isotonic saline solution and blood. Base excess was adjusted to 0 ± 5 mM with sodium hydrogen carbonate (8.4% (w/v), Fresenius, Germany).

### Test Loop Handling

All test loops were primed with isotonic saline solution and de-aired before being filled with blood. To yield a hematocrit of 30 ± 2%, excess saline solution was drained and replaced with blood. The target volume was 160 ± 5 mL with 1% accepted deviation between the mini test loops and the static reference. After a second de-airing, all test loops were run for 5 minutes to mix completely before verification and adjustment of hematocrit and base excess. For similar pre-pumping conditions, the corresponding flow rate of 2.4 ± 0.0 L min^−1^ of the limiting pump’s minimal speed was set for both test loops. After pre-pumping sampling, flow rate and pressure head were adjusted to the ASTM operating point (5 ± 0.25 L min^−1^ and 100 ± 3 mmHg, respectively^1^) and timers were started for further sampling.

A 160-mL static reference reservoir was similarly prepared and kept at static conditions in a water bath.

### Blood Sampling

After 0.5 mL discard, seven 2.5-mL samples were taken before starting the operating point (pre-pumping sample) and then every 60 minutes for 6 hours onwards. Samples were directly assessed for blood count, blood gas and activated clotting time and processed to platelet-poor plasma in 3.2%-tri-sodium-citrate tubes (Sarstedt, Germany). Plasma samples were stored at − 20 to − 80 °C according to standard clinical practice and manufacturer’s instructions until further analysis.

### Cleaning Procedure

Re-usage of blood pumps requires thorough cleaning of all parts in contact with blood. To ensure removal of cells and proteins, all pumps were first rinsed thoroughly with tap water until effluent was visually clear. The pumps were then set up in a cleaning-in-place (CIP) loop^27^ and run with a pepsin/citrate solution for 1 hour followed by 15 minutes rinsing with de-ionized water. Pumps were then incubated in 1% (v/v) instrument disinfectant solution (Bomix plus, Bode Chemie, Germany) for 15 minutes and finally rinsed with de-ionized water for 30 minutes. Pumps were then dried overnight by filtered compressed air and boxed until the next experiment.

### Analysis of Hemolysis

Analysis of hemolysis was performed according to DIN 58931:2010-08^6^ by means of the cyanmethemoglobin (HiCN) method. In brief, plasma samples were thawed in a water bath at 37 °C for 8 minutes and diluted 1:5 (v/v) with HiCN conversion solution (fHb (HiCN), Bioanalytic GmbH, Germany) in duplicates in standard micro cuvettes (Brandt, Germany). After incubation, converted plasma free hemoglobin (pfHb) was photometrically detected at 540 nm with 680 nm reference wavelength. Duplicate results were accepted with a coefficient of variation (CV) ≤ 0.05. Hemolysis is presented as ΔpfHb (mg dL^−1^) and modified and normalized milligram index of hemolysis (MIH and mgNIH, respectively), with

1 $$ \Delta {\text{pfHb}}_{t} = {\text{pfHb}}_{t} - {\text{pfHb}}_{{{\text{pre}}}} , $$

2 $$ {\text{MIH}} = \frac{{\Delta {\text{pfHb}}_{t}  \times \left( {100 - {\text{Hct}}_{t} } \right){\text{ }}/100}}{{{\text{Hb}}_{{{\text{pre}}}} }} \times \frac{{10^{6} }}{{\frac{{Q_{t}  \times T}}{{V_{t} }}}},  $$

3 $$ {\text{mgNIH}} = \frac{{\Delta {\text{pfHb}}_{t} \times \left( {100 - {\text{Hct}}_{t} } \right)}}{100} \times \frac{100 }{{\frac{{Q_{t} \times T}}{{V_{t} }}}}, $$

4 $$ \frac{{Q_{t} \times T}}{{V_{t} }} = \# {\text{passages}}_{t } = 60 \times \mathop \sum \limits_{i = 60 }^{t} \frac{{Q_{i} }}{{V_{i} }}\quad {\text{with}}\quad \left( {t = 60, 120, 180, 240, 360} \right); $$

with ΔpfHb_t_: Increase of plasma free hemoglobin (mg L^−1^) in the sampling interval, Hb_pre_: Pre-pumping total hemoglobin (mg L^−1^), Hct_*t*_: Hematocrit (%), *Q*_*t*_: Flow rate (L min^−1^), *V*_*t*_: Test loop volume (L), *T*: Elapsed time (min) and *#passages*: Absolute number of pump-passages

As previously recommended,^27^ the number of pump-passages in Eqs. (2) and (3) is corrected for the change of volume and flow rate over the sampling intervals by means of Eq. (4).

### Analysis of vWF Degradation

vWF concentration (vWF:Ag) and collagen-binding functionality (vWF:CB) were analyzed by commercial enzyme-linked immunosorbent assay (ELISA) kits (vWF:Ag ELISA and vWF:CB ELISA Collagen Type I, both Technozym, Germany).Duplicate results were accepted with a CV ≤ 0.10 for vWF:Ag and ≤ 0.13 for vWF:CB.

Ratios of vWF:CB and vWF:Ag were calculated for each sample with a cut-off for clinically relevant vWF degradation of 0.80^25^ and were depicted as absolute and normalized (ΔvWF-) ratios with

5 $$ \Delta {\text{vWF-ratio}}_{t} = {\text{vWF-ratio}}_{t} - {\text{vWF-ratio}}_{{{\text{pre}}}} ; $$

with ΔvWF-ratio_t_: Decrease of vWF-ratio in the sampling interval.

### Statistical Analysis

Statistical analysis was performed with Prism 9 (GraphPad, USA). Results were analyzed for each group, and normal distribution was verified with Shapiro Wilks test. Outliers were identified if below or above twice the interquartile range (IQR) or by means of the ROUT method^15^ with a maximum false discovery rate of 0.5%. Normally-distributed continuous variables are depicted as mean ± standard deviation (SD) or with 95% confidence interval (CI). Comparison within the groups were performed with mixed-effects analysis with Geisser-Greenhouse correction and Dunnett’s correction for multiple comparisons. For comparison between the groups, ANOVA or mixed-effects analysis with Geisser-Greenhouse correction and Tukey’s multiple comparison correction or *t*-test were used, as appropriate. An adjusted exact *p*-value was considered significant with *p *≤ 0.05 (*).

## Results

Mean technical and baseline hematologic parameters of mini test loops and the static reference are depicted in Tables 1 and 2. Flow rate, pressure difference and number of pump passages did not differ significantly between the test loops. Temperature of both mini test loops was significantly lower than of the static reference and not ASTM-conform but re-stabilized to 36.8 ± 0.5 and 37.1 ± 0.5 °C within the first 60 minutes for BPX-80 and HM3, respectively. Mean baseline blood volume and hematologic parameters did not differ significantly between the test loops and the static reference.

**Table 1:** Technical parameters of mini test loops.

Variable; mean ± SD	BPX-80 mini	HM3 mini	Static reference	Overall *p*-value	BPX-80 vs HM3 *p*-value
Volume (mL) at start of operating point	160.6 ± 0.4	160.6 ± 0.4	160.7 ± 0.3	0.985	0.998
Flow (L min^-1^) at start of operating point	5.04 ± 0.06	5.05 ±0.08	–	–	0.840
Pressure difference (mmHg) at start of operating point	100 ± 0	99 ± 1	–	–	0.114
Temperature (°C) at start of operating point	31.2 ± 3.8	32.7 ± 2.5	36.9 ± 0.2	< 0.001	0.459
Number of passages through pump at 360 min	12306 ± 54	12336 ± 108	–	–	0.467

**Table 2:** Baseline human hematologic parameters of mini test loops.

Variable, mean ± SD	BPX-80 mini	HM3 mini	Static Reference	Overall *p* value	BPX-80 vs HM3 *p* value
Hematocrit (%)	30.4 ± 1.1	30.2 ± 1.2	30.5 ± 1.1	0.890	0.954
Red blood cells (10^6^ µL^−1^)	3.59 ± 0.10	3.57 ± 0.17	3.58 ± 0.09	0.967	0.967
Total hemoglobin (g dL^−1^)	10.61 ± 0.56	10.58 ± 0.60	10.64 ± 0.45	0.967	0.991
White blood cells (10^3^ µL^−1^)	3.78 ± 0.78	3.75 ± 0.78	3.70 ± 0.81	0.973	0.996
Platelets (10^3^ µL^−1^)	160.67 ± 34.16	164.89 ± 29.32	164.22 ± 32.19	0.956	0.958
pH	7.40 ± 0.03	7.39 ± 0.04	7.36 ± 0.03	0.084	0.933

### Sample Exclusion

Seven samples (ΔpfHb) of two experiments with the BPX-80 mini test loop, 1 sample of the HM3 mini test loop (vWF-ratio) and 2 samples of the static reference (ΔpfHb and ΔvWF-ratio) were identified as outliers, respectively. The identified outliers of the BPX-80 mini test loop were most probably caused by overheating events or insufficient de-airing during the filling process with impact on increasing pfHb, and we thus excluded these test loops from statistical analysis of hemolysis. The outliers of the HM3 loop and the static reference were most probably caused by mistakes during sampling or analysis and respective samples were excluded from statistical analysis. Furthermore, in one experiment, we observed pre-pumping pfHb values ≥ 20 mg dL^−1^ in both mini loops and the static reference and thus excluded that experiment from statistical analysis of hemolysis.^1^

### Assessment of Hemolysis

**Figure 2:**
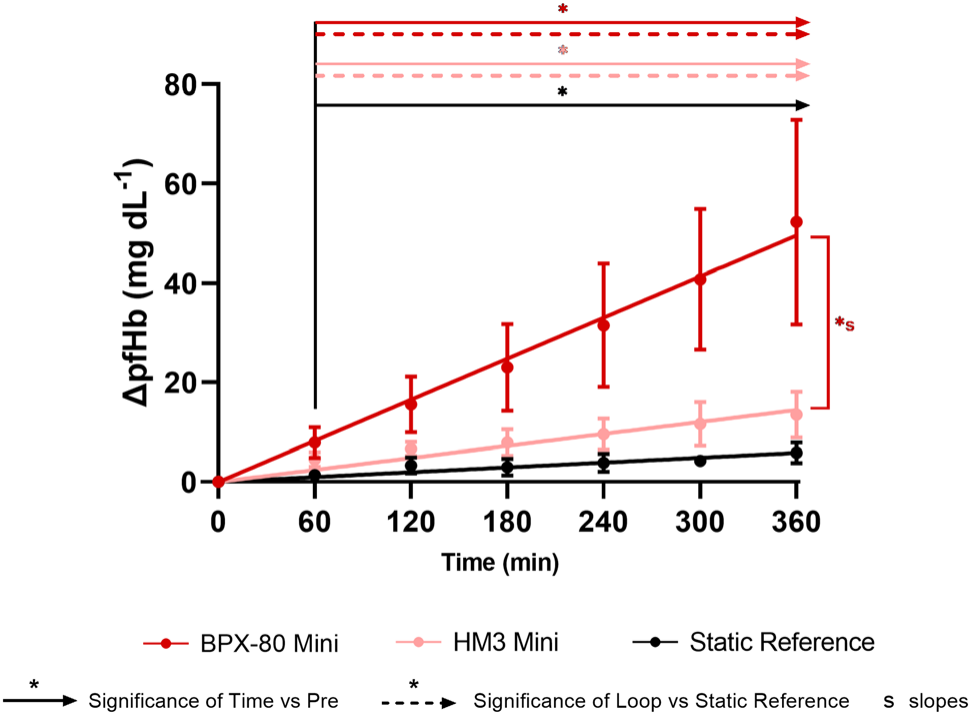
Longitudinal profiles of ΔpfHb of BPX-80 (dark red) and HM3 (light red) mini test loops and static reference (black) are depicted as mean ± SD with mean linear regression and significance of time *vs* pre (straight lines), consistent significance of loop *vs* static reference (dashed lines) and significant differences between the slopes (s).

Mean pre-pumping pfHb was ≤ 17 mg dL^−1^ and not significantly different for all test loops and static reference (Table 3). Mean ΔpfHb significantly increased over time in both mini test loops and the static reference (Fig. 2 and Table 4). However, compared to the static reference, ΔpfHb in both mini test loops was significantly higher from 60 minutes onwards and mean slopes were significantly steeper for BPX-80 and HM3 with a factor of 8.5 and 2.5, respectively (Fig. 2 and Tables 4 and 5).

**Table 3:** Descriptive data of pre-pumping human plasma free hemoglobin (mg dL^−1^) of mini test loops.

Loop	Mean pfHb (mg dL^−1^)	SD (mg dL^−1^)	*N* valid	*N* missing	BPX-80 vs HM3 *p* value	Loop vs static reference
BPX-80 mini	16.30	2.29	8	1	0.943	0.502
HM3 mini	16.63	1.73	8	1	–	0.328
Static Reference	15.18	1.87	8	1	–	–

**Table 4:** Descriptive data of human ∆plasma free hemoglobin (mg dL^−1^) of mini test loops.

Loop	Time (min)	Mean ΔpfHb (mg dL^−1^)	SD (mg dL^−1^)	*N* valid	*N* missing	Time vs Pre *p* value	BPX-80 vs HM3 *p* value	Loop vs static reference p value
BPX-80 mini	Pre	0.00	0.00	6	3	–	–	–
	60	7.93	3.09	6	3	0.006	0.091	0.006
	120	15.58	5.64	6	3	0.004	0.024	0.006
	180	23.05	8.75	6	3	0.005	0.016	0.005
	240	31.49	12.39	6	3	0.006	0.016	0.006
	300	40.79	14.12	6	3	0.003	0.007	0.003
	360	52.33	20.66	6	3	0.006	0.012	0.006
HM3 mini	Pre	0.00	0.00	8	1	–	–	–
	60	4.48	1.50	8	1	< 0.001	–	0.001
	120	6.62	1.45	8	1	< 0.001	–	0.002
	180	7.89	2.69	8	1	< 0.001	–	0.002
	240	9.57	3.11	8	1	< 0.001	–	0.002
	300	11.63	4.41	8	1	< 0.001	–	0.005
	360	13.50	4.61	8	1	< 0.001	–	0.004
Static reference	Pre	0.00	0.00	8	1	–	–	–
	60	1.39	1.01	8	1	0.025	–	–
	120	3.32	1.66	8	1	0.003	–	–
	180	2.94	1.68	8	1	0.007	–	–
	240	3.83	1.79	8	1	0.002	–	–
	300	4.22	1.08	8	1	< 0.001	–	–
	360	5.84	2.05	7	2	0.001	–	–

**Table 5:** Descriptive data of linear regression of human Δplasma free hemoglobin of mini test loops.

Loop	Mean *R*^2^ ΔpfHb	Mean slope ΔpfHb	95% CI	*N* valid	*N* missing	BPX-80 vs HM3 *p* value	Loop vs static reference *p* value
BPX-80 mini	0.99	0.1377	0.1311 to 0.1444	6	3	< 0.001	< 0.001
HM3 mini	0.93	0.0401	0.0348 to 0.0453	8	1	–	< 0.001
Static Reference	0.89	0.0162	0.0134 to 0.0189	8	1	–	–

**Figure 3:**
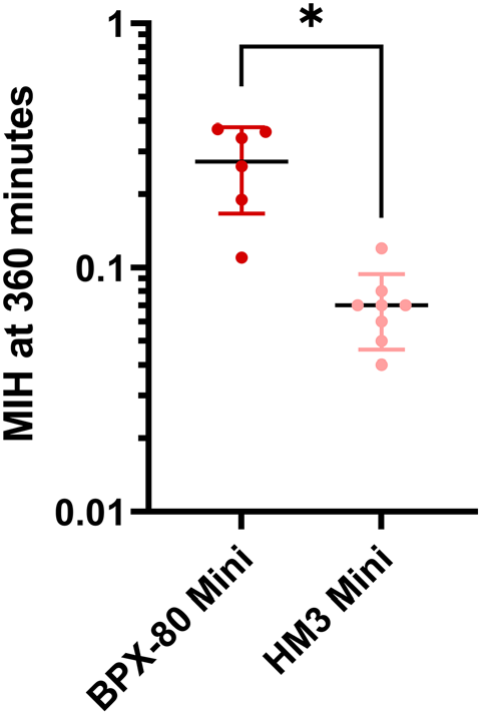
Modified index of hemolysis (MIH) at 360 minutes of BPX-80 and HM3 mini test loops depicted as mean ± SD.

**Figure 4:**
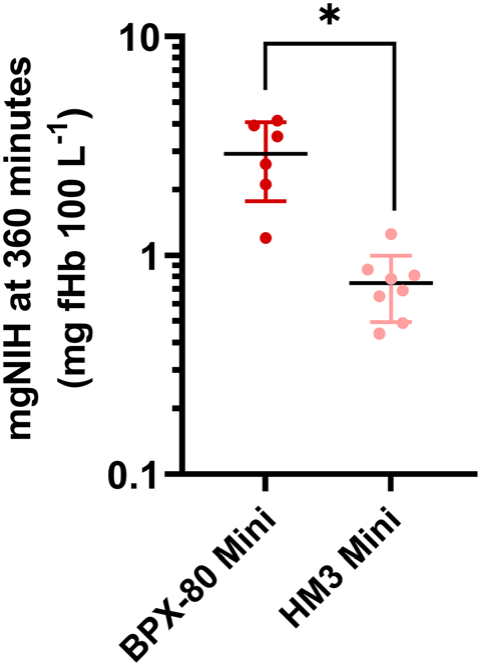
Normalized Milligram index of hemolysis (mgNIH) at 360 minutes of BPX-80 and HM3 mini test loops depicted as mean ± SD.

Between the pumps, BPX-80 generated a 3.4-fold higher mean increase in ΔpfHb than HM3 (Fig. 2 and Tables 4 and 5). Correspondingly, with correction for volume, flow, hematocrit and total hemoglobin, mean MIH (Fig. 3 and Table 6) and mgNIH (Fig. 4 and Table 7) at 360 minutes were significantly higher for BPX-80 compared to HM3.

**Table 6:** Human modified index of hemolysis of mini test loops at 360 minutes.

Loop	Mean MIH at 360 min	SD	N valid	N missing	BPX-80 vs HM3 *p* value
BPX-80 mini	0.27	0.10	6	3	< 0.001
HM3 mini	0.07	0.02	8	1	–

**Table 7:** Human normalized milligram index of hemolysis of mini test loops at 360 minutes.

Loop	Mean mgNIH at 360 min (mg pfHb 100 L^−1^)	SD (mg pfHb 100 L^−1^)	*N* valid	*N* missing	BPX-80 vs HM3 *p* value
BPX-80 mini	2.92	1.15	6	3	< 0.001
HM3 mini	0.75	0.25	8	1	–

### Assessment of vWF Degradation

**Figure 5:**
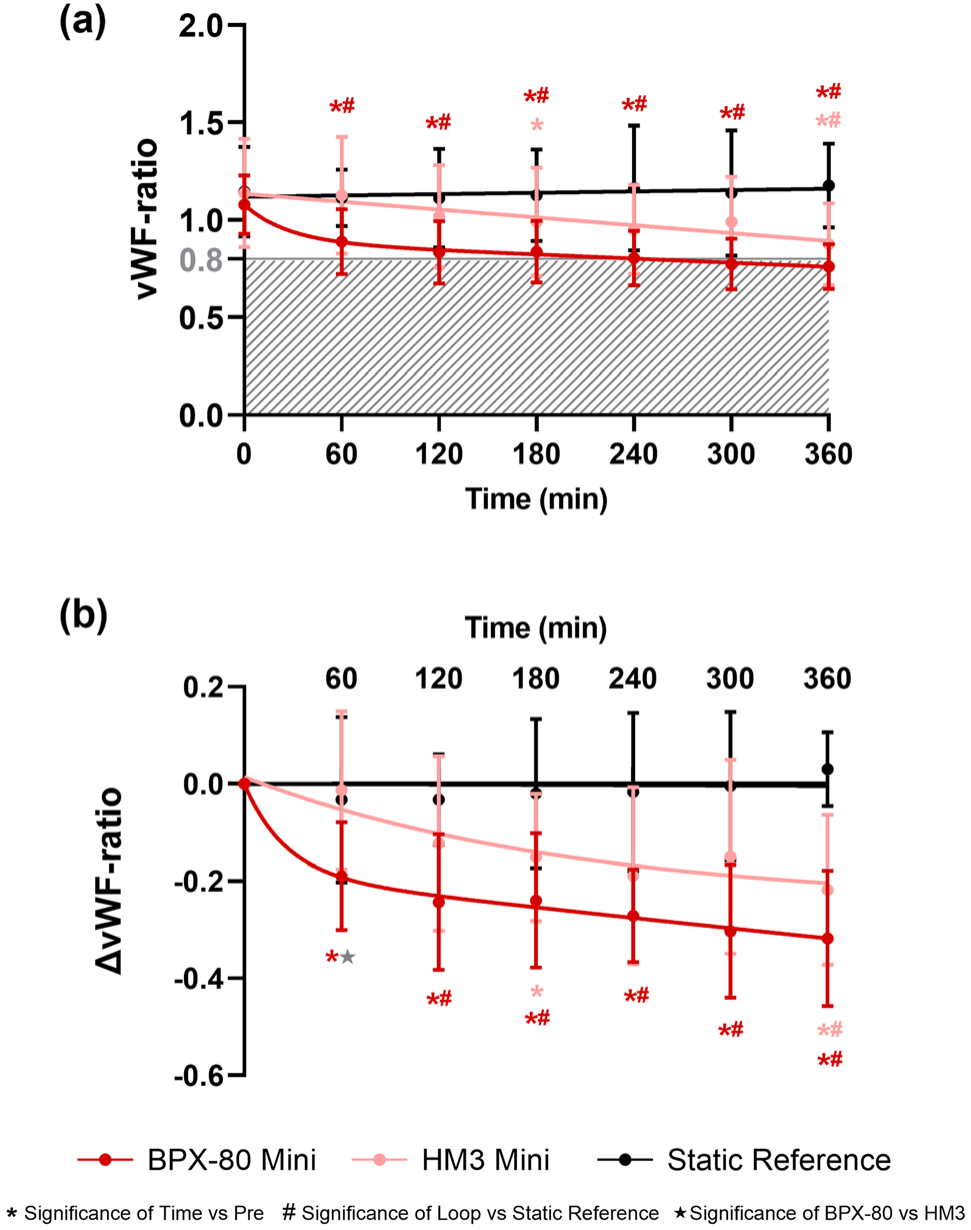
Longitudinal profiles of vWF-ratio (a) and ΔvWF-ratio (b) of BPX-80 (dark red) and HM3 (light red) mini test loops and static reference (black) are depicted as mean ± SD with mean regression and significance of time vs pre (*), significance of loop vs static reference (#) and significance of BPX-80 vs HM3 (★). The shaded area in (a) marks the clinically relevant range with pathological vWF-ratios ≤ 0.80.

Mean pre-pumping vWF-ratios were not significantly different and in normal range of > 0.80 for both mini loops and the static reference (Fig. 5a and Table 8). Within both mini loops but not the static reference, vWF-ratios significantly decreased over time. However, pathologic vWF-ratios < 0.80 clinically associated with an acquired von Willebrand syndrome (AvWS) were only observed with BPX-80 from 300 min onwards (Fig. 5a and Table 8). In line with those findings, decrease in mean ΔvWF-ratios was stronger in the BPX-80 than in the HM3 test loop, (Fig. 5b and Table 9). Compared to the static reference, vWF-ratios as well as ΔvWF-ratios decreased significantly in both mini loops (Fig. 5 and Tables 8 and 9).

**Table 8:** Descriptive data of human vWF-ratio of mini test loops.

Loop	Time (min)	Mean vWF-ratio	SD	*N* valid	*N* missing	Time vs pre *p* value	BPX-80 vs HM3 *p* value	Loop vs Static Reference *p* value
BPX-80 mini	Pre	1.08	0.15	9	0	–	0.833	0.746
	60	0.89	0.17	9	0	0.004	0.135	0.020
	120	0.83	0.16	9	0	0.003	0.207	0.036
	180	0.84	0.16	9	0	0.004	0.354	0.022
	240	0.80	0.14	9	0	< 0.001	0.258	0.026
	300	0.77	0.13	9	0	< 0.001	0.073	0.024
	360	0.76	0.12	9	0	< 0.001	0.381	< 0.001
HM3 mini	Pre	1.14	0.28	9	0	–	–	0.998
	60	1.13	0.30	9	0	> 0.999	–	0.993
	120	1.02	0.26	9	0	0.283	–	0.724
	180	0.99	0.28	9	0	0.041	–	0.520
	240	0.95	0.23	9	0	0.066	–	0.269
	300	0.99	0.23	9	0	0.210	–	0.519
	360	0.88	0.21	8	1	0.010	–	0.027
Static reference	Pre	1.15	0.23	9	0	–	–	–
	60	1.11	0.15	9	0	0.979	–	–
	120	1.11	0.25	9	0	0.793	–	–
	180	1.13	0.24	9	0	0.996	–	–
	240	1.16	0.32	9	0	0.999	–	–
	300	1.14	0.32	9	0	> 0.999	–	–
	360	1.18	0.21	9	0	0.699	–	–

**Table 9:** Descriptive data of human ΔvWF-ratio of mini test loops

Loop	Time (min)	Mean ΔvWF-ratio	SD	*N* valid	*N* missing	Time vs pre *p* value	BPX-80 vs HM3 *p* value	Loop vs static reference *p* value
BPX-80 mini	Pre	0.00	0.00	9	0	–	–	–
	60	− 0.19	0.11	9	0	0.004	0.044	0.085
	120	− 0.24	0.14	9	0	0.004	0.277	0.006
	180	− 0.24	0.14	9	0	0.004	0.364	0.015
	240	− 0.27	0.10	9	0	< 0.001	0.476	0.007
	300	− 0.30	0.14	9	0	< 0.001	0.176	0.001
	360	− 0.32	0.14	9	0	< 0.001	0.367	< 0.001
HM3 mini	Pre	0.00	0.00	9	0	–	–	–
	60	− 0.01	0.16	9	0	> 0.999	–	0.969
	120	− 0.12	0.18	9	0	0.264	–	0.405
	180	− 0.15	0.13	9	0	0.035	–	0.158
	240	− 0.19	0.18	9	0	0.059	–	0.132
	300	− 0.15	0.20	9	0	0.199	–	0.225
	360	− 0.22	0.15	8	1	0.019	–	0.005
Static Reference	Pre	0.00	0.00	9	0	–	–	–
	60	− 0.03	0.17	9	0	0.979	–	–
	120	− 0.03	0.09	9	0	0.798	–	–
	180	− 0.02	0.15	9	0	0.996	–	–
	240	− 0.02	0.16	8	1	0.999	–	–
	300	0.00	0.15	9	0	> 0.999	–	–
	360	0.03	0.08	9	0	0.710	–	–

## Discussion

In this study, we validated our recently verified mini test loop for the assessment of LVAD-related hemolysis and vWF degradation for the paired testing of two pumps and a static reference with a single donation of 450 mL of fresh human blood.

### Assessment of Hemolysis

Consistent with our previous findings,^27^ the BPX-80 pump led to significantly higher pfHb, mgNIH and MIH than the HM3. Interestingly, both pumps generated less hemolysis than previously observed with porcine blood.^27^ Although slaughterhouse blood is a widely used and valid option to assess hemolysis by LVADs,^1^ it can be more stressed and thus more susceptible to damage.^28^ As lactate concentration, a (pre-slaughter) stress marker,^10^,^28^ was significantly higher in our previous study, the porcine red blood cells could have had a lower critical shear rate or exposure time limit or higher sensibility to further parameters and hence been more prone to hemolysis.^8^ Moreover, in this study, the pH was closer to physiological and better maintained over time, which might have also accounted to the lower hemolysis in human blood. However, with correction for volume, flow rate, hematocrit and total hemoglobin, mean differences between porcine and human MIH are consistent for both pumps, and mean differences in MIH between BPX-80 and HM3 are thus preserved with the mini loop for either species. Moreover, as the mean difference between the pumps is also consistent with our previous results for the original 450-mL ASTM loops with porcine blood, we recommend our mini loops as valid option for the ASTM-conform preclinical assessment of hemolysis with only one single donation of human blood.

So far, no other studies exist that used human blood in a small test loop set-up with BPX-80 or HM3 and the same test duration and operating point. Thus, comparison of our results to literature data is always flawed to some extent. Zayat *et al*. observed unusually high pfHb with human blood in a small HM3 test loop (82 mL); however, this is probably due to a different shear profile resulting from a different test loop set-up and an unusual low-flow operating point of ≤ 0.1 L min^−1^.^29^

With a more similar set-up to ours, but with an almost double-sized test loop (300 mL) adapted from Olia *et al*.,^19^ Berk *et al*. recently compared HVAD or HMII against a device under test (CH-VAD) and reported mgNIH values of 5.25 mg pfHb 100 L^−1^ for HVAD, 5.83 mg pfHb 100 L^−1^ for HMII and 1.35 mg pfHb 100 L^−1^ for CH-VAD after 4 hours at a flow rate of 4.5 L min^−1^.^3^ As HM3 has a better shear profile than HMII and HVAD,^24^,^26^ we consider our lower mgNIH results for HM3 to be in line with those findings, although the pressure differences varied between the studies. When compared to literature data of clinical studies, the low *in vitro* hemolysis profile of HM3 in our mini loop with human blood might well reflect absent clinically relevant hemolysis reported for HM3 patients.^13^,^18^,^22^ However, also subclinical levels of hemolysis can trigger cascades leading to severe adverse events such as thrombosis and stroke and should thus not be underestimated.^20^

In comparison with the loop from Berk *et al*.,^3^ the smaller volume of our mini loops (160 mL) beneficially permits the paired testing of (at least) 2 pumps and 1 static reference even at the recent target hematocrit of 35% as required by the international standard for biological evaluation of medical devices (10993-4)^7^ and ASTM F1841^1^,^2^ with only one single donation of human blood. Blood donations of 500 mL and ≥ 47% hematocrit will even allow for the direct comparison of 4 identical test loops, e.g. 3 different pumps plus 1 static reference, 3 different operating points plus 1 static reference or 2 different medications or blood treatments each with 1 respective static reference. Additionally, the volume of taken samples also allows for analyzing additional factors such as markers for activation of platelets, complement system or coagulation.

### Assessment of vWF Degradation

Compared to pre-pumping values, significant vWF degradation occurred in all test loops but not in the static reference. Consistent with the hemolysis results, vWF degradation was stronger in the BPX-80 loops and not clinically relevant in the HM3 test loop. As seen for pfHb increase, both test loops with human blood showed less vWF degradation than observed in our previous study,^27^ which might be due to more stressed and thus more susceptible porcine slaughterhouse blood therein. However, differences in mean ΔvWF-ratios of mini loops with BPX-80 and HM3 were similar between both species.

As discussed above, data of well comparable *in vitro* set-ups, i.e. usage of fresh human blood (in a small test loop) at a similar operating point and assessment of vWF functionality, are limited in current literature. Zayat *et al*. reported pathological decrease in vWF-ratios based on platelet-binding activity in HM3 loops with human blood.^29^ In line with the hemolysis results, we observed less decrease in vWF-ratios based on collagen-binding activity. However, as mentioned before, this might be due to distinctive differences between the loop set-ups, operating points and resulting shear profiles.

In a loop adapted from Olia *et al*.,^19^ Chen *et al*. observed decreased high molecular weight multimers (HMWM) of vWF in human blood circulated with a Centrimag pump at 4.5 L min^−1^ and 150 mmHg pressure difference.^4^ Although reduced HMWM cannot be directly translated into vWF-ratios assessed by (collagen-binding) functionality, we assume our results to be in line with those findings, as we also observed decreased vWF-ratios with both pumps and vWF-ratios < 0.80 in the BPX-80 loop.

### Limitations

Our results underlie general limitations of smaller studies; however, we provide more valid experiments than recommended by the ASTM.^1^,^2^ As in our previous study,^16^ few discrepancies from the ASTM standard exist in our set-up: Our institutional heparin dosage is higher than recommended and could have affected platelet activation or hemolysis.^9^ The test loops and static reference were primed with isotonic saline solution instead of phosphate buffered solution (PBS) and thus lacked the buffer capacity comprised by PBS. Nevertheless, pH of the human blood was more physiological and better maintained as in the slaughterhouse blood of our previous study^27^ and might thus be of less concern when testing with fresh donations of human blood.

### Conclusion

In this study, we validated our mini test loop for the ASTM-conform assessment of blood damage with paired testing of at least two LVADs and a static reference using only one standard single donation of fresh human blood of 450 mL. Differences in mean human MIH and in mean normalized vWF-ratio between BPX-80 and HM3 were consistent with our previous results with porcine blood. Our mini loop is thus valid for ASTM-conform preclinical testing of LVADs with fresh, non-pooled human blood, and the small total volume of only 160 mL of our mini loop will also allow for such testing at higher hematocrits ≥ 35%. Our mini loop may thus add to a better translation of device-related human blood damage from *in vitro* to *in vivo* and contribute to further optimization of blood pumps for future LVAD patients.
